# Homozygosity for the E526V Mutation in Fibrinogen A Alpha-Chain Amyloidosis: The First Report

**DOI:** 10.1155/2015/919763

**Published:** 2015-06-23

**Authors:** Isabel Tavares, Luísa Lobato, Carlos Matos, Josefina Santos, Paul Moreira, Maria João Saraiva, António Castro Henriques

**Affiliations:** ^1^Department of Nephrology, Centro Hospitalar de São João, 4200-319 Porto, Portugal; ^2^Nephrology and Infectious Diseases Research and Development Group, INEB (I3S), University of Porto, 4150-180 Porto, Portugal; ^3^Department of Nephrology, Centro Hospitalar do Porto, Hospital de Santo António, 4099-001 Porto, Portugal; ^4^Unit for Multidisciplinary Research in Biomedicine, Instituto de Ciências Biomédicas Abel Salazar, University of Porto, 4050-313 Porto, Portugal; ^5^Molecular Neurobiology, Institute of Molecular and Cellular Biology, University of Porto, 4150-180 Porto, Portugal

## Abstract

Systemic hereditary amyloidoses are autosomal dominant diseases associated with mutations in genes encoding ten different proteins. The clinical phenotype has implications on therapeutic approach, but it is commonly variable and largely dependent on the type of mutation. Except for rare cases involving gelsolin or transthyretin, patients are heterozygous for the amyloidogenic variants. Here we describe the first patient identified worldwide as homozygous for a nephropathic amyloidosis, involving the fibrinogen variant associated with the fibrinogen alpha-chain E526V (p.Glu545Val) mutation. In 1989, a 44-year-old woman presented with hypertension, hepatosplenomegaly, nephrotic syndrome, and renal failure. She started hemodialysis in 1990 and 6 years later underwent isolated kidney transplantation from a deceased donor. Graft function and clinical status were unremarkable for 16 years, despite progressively increased left ventricular mass on echocardiography. In 2012, 4 months before death, she deteriorated rapidly with severe heart failure, precipitated by *Clostridium difficile* colitis and urosepsis. Affected family members developed nephropathy, on average, nearly three decades later, which may be explained by the gene dosage effects on the phenotype of E526V (p.Glu545Val) fibrinogen A alpha-chain amyloidosis.

## 1. Introduction

Hereditary fibrinogen A alpha-chain (AFib) amyloidosis is a systemic amyloid disease first characterized in 1993 in a Peruvian kindred [1–4]. It presents with proteinuria and features a progressive decline in kidney function to end stage renal failure (ESRF) within 5 years of diagnosis [3]. Nonetheless, there is a wide variability in disease onset, systemic involvement, and penetrance. Renal replacement therapy and transplantation are currently the mainstay of therapy. However, because the liver is the source of the amyloidogenic variant fibrinogen and there is no evidence that WT fibrinogen can be amyloidogenic, the only curative treatment is liver transplantation [5].

AFib amyloidosis appears to be more common worldwide than previously recognized [6]. The R554L (p.Arg573Leu) mutation was the first fibrinogen amyloidogenic variant identified [1]. To date, thirteen amyloidogenic mutations have been reported in the fibrinogen alpha-chain gene (*FGA*) (http://amyloidosismutations.com/mut-afib.php), accounting for 8% of hereditary amyloidosis cases [7]. The most common mutant variant, E526V (p.Glu545Val), was identified heterozygously in kindred members of Irish, British, Polish, Portuguese, French, German, and Brazilian origin [2, 3, 8–11]. Homozygosity has only been reported for hereditary gelsolin and transthyretin amyloidosis (Table 1). In gelsolin (AGel) amyloidosis, homozygotes have been reported to show earlier onset and more severe clinical manifestations than heterozygotes, explained by the lethal effects of the mutant genes [12, 13]. However, in transthyretin (ATTR) amyloidosis, the underlying molecular mechanisms are largely unknown [14–23].

Here, we report the first homozygous patient with AFibE526V (p.Glu545Val) amyloidosis, identified in 1989 and followed up for 23 years. In this reported kindred, the comparison of the clinical pictures of homozygote and heterozygotes provides important information about the gene dosage effects on the phenotype of AFib amyloidosis.

## 2. Case Report

### 2.1. Proband

**Figure 1:**
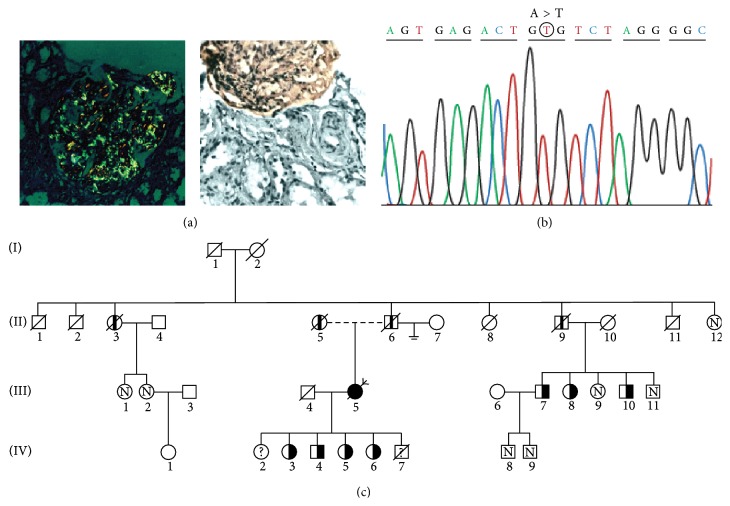
Homozygous E526V (p.Glu545Val) mutation in the fibrinogen alpha-chain gene (*FGA*) associated with fibrinogen A alpha-chain amyloidosis in a Portuguese patient. (a) shows abundant glomerular amyloid deposition with typical apple-green birefringence (Congo red staining under polarized light, ×200, left). Immunohistochemical staining was positive with polyclonal anti-fibrinogen antibodies, (×200, right). (b) shows a partial sequence chromatogram of* FGA*. The mutation identified in the proband, which alters codon 545 (position 526 of the mature protein) from GAG (glutamic acid) to GTG (valine), is depicted in a circle. (c) shows the pedigree of the affected kindred. The homozygous patient (proband) is indicated by the arrow. The* FGA* p.Glu545Val mutation was identified heterozygously in family members III7, III8, III10, IV3, IV4, IV5, and IV6 (indicated by half-solid symbols). Obligatory heterozygotes IV2 and IV7 (indicated by question marks) did not perform genotyping because the former was abroad and the latter died at young age. Those with chronic renal failure who have not undergone histologic or genetic testing are indicated by a black column inside the symbol. Familiars whose genetic tests were negative are indicated by an N inside the symbol. Blank symbols indicate that tests have not been conducted and/or information is unavailable for these individuals. Slashes denote deceased members.

A 44-year-old Caucasian northern Portuguese woman who had suffered a single previous episode of upper gastrointestinal hemorrhage presented with hypertension, nephrotic syndrome, and renal failure in April 1989. Renal biopsy 1 year later revealed abundant glomerular amyloid deposition (Figure 1(a)); her serum creatinine was 4.0 mg/dL and proteinuria of 7.5 g/day was detected. In the absence of any underlying inflammatory disease and unawareness of family history, she was presumed to have immunoglobulin light chain (AL) amyloidosis. However, there was no evidence of cardiac amyloidosis, and neither a monoclonal immunoglobulin nor a plasma cell disorder was identified. She was treated with melphalan and corticosteroids but progressed rapidly to ESRF and started hemodialysis in December 1990. During hemodialysis, her functional status remained good.

Successful isolated renal transplantation (RTx) was performed in August 1996. At the time, she had no other health problems. Physical examination revealed an enlarged liver and spleen. There was no anemia or thrombocytopenia and liver function tests were normal. Blood coagulation tests revealed low fibrinogen levels. Echocardiography showed mild left ventricular hypertrophy without the typical speckle appearance. Abdominal ultrasonography showed hepatomegaly (17.5 cm), splenomegaly (14.5 cm), and bilateral kidney atrophy. A neurophysiologic study of the lower limbs found mild sensory peripheral neuropathy. Induction and maintenance immunosuppression for transplantation involved corticosteroids, cyclosporine, and azathioprine.

### 2.2. Histology and Immunohistochemistry

In 2001, we began an extensive investigation to determine the patient's amyloid type. Congo red staining of 6 *μ*m formalin fixed, paraffin embedded section of the native kidney biopsy confirmed abundant glomerular amyloid deposition and absence of vascular and interstitial involvement. Immunohistochemical staining was performed on 2 *μ*m sections of amyloid-containing tissue using standard methods and a rabbit/mouse, peroxidase/diaminobenzidine detection system (REAL EnVision: Dako, Glostrup, Denmark). mAbs were directed against serum amyloid A (Dako), apolipoprotein A-II (Abcam, Cambridge, UK), and transthyretin [24]; polyclonal antibodies were used for kappa light chain, lambda light chain, fibrinogen A alpha-chain (FGA), transthyretin, apolipoprotein A-I, and lysozyme (Dako). For light chains and FGA, sections were treated with 10 *μ*g/mL proteinase K for 10 min at 37°C and 10 min at room temperature. Blocking was performed with 5% bovine serum albumin/phosphate buffered saline (BSA/PBS). Sections were incubated with the antibodies for 2 h at room temperature and diluted in 1% BSA/PBS as follows: monoclonal anti-transthyretin, used directly; polyclonal anti-transthyretin, 1 : 500; anti-serum amyloid A, 1 : 100; anti-kappa light chain, 1 : 1000; anti-lambda light chain, 1 : 2000; anti-FGA, 1 : 800; anti-lysozyme, 1 : 300; anti-apolipoprotein A-I, 1 : 400; and anti-apolipoprotein A-II, 1 : 600. Positive control tissues containing these amyloid proteins were also stained during each run. The glomerular amyloid deposits in the patient's kidney biopsy reacted with the anti-serum to FGA (Figure 1(a)).

### 2.3. Genetic Evaluation

DNA was extracted from peripheral white blood cells obtained from whole blood. Exon 5 of the* FGA* gene was amplified by the polymerase chain reaction (PCR) with primers flanking the coding region (forward 5′-CCT TCT TCG ACA CTG CCT CAA CTG-3′ and reverse 5′-TCC TCT GTT GTA ACT CGT GCT-3′), which amplified a fragment of 224 base pairs encompassing nucleotide 4827 to 5051. PCR products were analyzed by agarose gel electrophoresis, purified, and sequenced with Big Dye Terminator Cycle Sequencing Kit (Applied Biosystems, Foster City, CA, USA) in an Applied Biosystems 3700 sequencer. Sequences were analysed using ChromasPro. A homozygous A to T transversion at nucleotide 4909 in* FGA* was detected; this changes codon 526 of the mature protein (corresponding to position 545 of the unprocessed gene product), from GAG, encoding glutamic acid, to GTG, encoding valine (Figure 1(b)). Codons are numbered according to reference sequence NM_000508.3.

### 2.4. Proband Outcome

During 16 years of follow-up after RTx, there were no episodes of rejection and she was normotensive. Maximal proteinuria of 0.9 g/day was detected in 2009 and renal graft function remained good until 4 months before her death; thus, no renal allograft biopsy was performed. Peptic ulcer due to* Helicobacter pylori* infection was diagnosed after an episode of upper gastrointestinal hemorrhage. Apparent progression of extrarenal amyloid disease was mainly cardiovascular and hepatic. Repeated electrocardiography found sinus rhythm, normal intervals, and no criteria for hypertrophy. Serial echocardiography showed left atrial enlargement (53 mm), severely abnormal left ventricular hypertrophy (left ventricular mass index increased from 154 g/m^2^ in 2006 to 170 g/m^2^ in 2012, with a reference range of 43–95 g/m^2^), mild degenerative valvular disease, preserved systolic ventricular function, and moderate pulmonary hypertension. These findings were consistent with cardiac amyloidosis. Hepatic involvement was characterized by mild elevations of gamma-glutamyl transferase and alkaline phosphatase with increasing hepatomegaly; the liver's ultrasound diameter reached 22.7 cm in September 2012. At that time, the spleen diameter was 9.4 cm and there were several calcifications. There was no sign of portal hypertension. Relevant laboratory data are listed in Table 2.

In August 2012, 4 months before her death, the patient was hospitalized for* Clostridium difficile* colitis, which was successfully treated with metronidazole. One month later, she was admitted for congestive heart failure with an N-terminal pro-B-type natriuretic peptide (NT-proBNP) level of 31 937 pg/mL; this was controlled with diuretic therapy. In November 2012, she was hospitalized for hyponatremia associated with effective circulating volume depletion. She died in December 2012 after admission for* Klebsiella pneumoniae* urosepsis, congestive heart failure with NT-proBNP 14 056 pg/mL unresponsive to diuretic therapy, and acute kidney allograft injury. Autopsy was not performed.

### 2.5. Kindred

After some years of research, a family tree was obtained (Figure 1(c)). The research protocol was approved by the Health Ethics Commission of Centro Hospitalar de São João. The mother of the proband (II5) presented with hypertension aged 67 years. She had a stroke at 76 years and died from chronic renal failure (CRF) 1 year later. The father (II6) had hypertension from the age of 55 years, significant cardiovascular disease (peripheral artery disease, ischemic cardiopathy), and CRF from 77 years, dying from pneumonia at 82 years. One paternal aunt (II3) died at 84 years due to hemorrhagic cerebrovascular accident. She had hypertension from the age of 59 years and CRF from 69 years and had started hemodialysis at 79 years. One paternal uncle (II9) had hypertension from the age of 61 years, significant cardiovascular disease (peripheral artery disease, ischemic cardiopathy), and CRF from 72 years, dying from uremia at 78 years. Three first cousins (III7, III8, and III10) had hypertension (from 57, 55, and 53 years, resp.) and carried the same amyloidogenic fibrinogen mutation. As patient III5 was homozygous for the* FGA* p.Glu545Val mutation, all her offspring are obligatory heterozygotes. This condition was confirmed by genotyping in all, except IV2, who was abroad, and IV7, who died at young age. Members IV3 and IV4 had hypertension from the age of 45 and 43 years, respectively.

## 3. Discussion

Here, we describe the first AFib amyloidosis patient homozygous for the E526V (p.Glu545Val) mutation and her long term outcome after isolated RTx. The unexpected etiology and outcome of this case highlight important aspects of the clinical management of systemic amyloidosis in general. Our patient's prolonged survival without treatment and the absence of an identifiable monoclonal plasma cell disorder led us to question the diagnosis of AL amyloidosis. Retrospective finding that the amyloid deposits in her kidney biopsy were derived from FGA and the complete concordance between the presence of the E526V (p.Glu545Val) and the development of amyloidosis indicated that this mutation was the cause of the disease in the proband's family. This approach ensured family screening.

Immunohistochemical classification of 102 northern Portuguese patients with amyloidosis diagnosed in native kidney biopsies disclosed 4 (3.9%) cases of AFib, including our proband. They were all from the same rural geographical area and belonged to apparently unrelated families [25]. In the case of our homozygous patient, both parents had CRF and consanguinity was not possible to prove, but they may share a common ancestor given the possibility of an endemic focus of the disease in their region. In this context, homozygosity proposal was based on DNA sequencing (Figure 1(b)).

Previously reported AFib amyloidosis phenotypes result from the heterozygous genotype. The effect of homozygosity on phenotype has been reported for patients with hereditary transthyretin [14–23] or gelsolin [12, 13] amyloidosis (Table 1), but this is the first report for AFib amyloidosis. Our patient presented at a relatively early age and during long term follow-up apparently developed a heavy disease burden with multisystem involvement. After RTx, serial echocardiography demonstrated increased wall thickness, despite normotension and normal graft function, consistent with cardiac amyloidosis. The main forms of amyloidosis that affect the heart are light chain and ATTR amyloidosis. Cardiac involvement of AFib amyloidosis was described in a cohort of 22 AFib patients [4], 52% had abnormal echocardiographic findings suggestive of amyloid cardiomyopathy, and 55% had parasympathetic dysfunction and risk of bradycardia. Coronary atherosclerosis was identified in 68%. In the present case, cardiac involvement in the setting of established AFib amyloidosis, with left ventricular hypertrophy on echocardiography and a relative low-voltage electrocardiogram, complicated by congestive heart failure refractory to standard medical therapy, was considered cardiac amyloidosis. Two of the family members developed atherosclerotic cardiovascular disease with no echocardiographic evidence of cardiac amyloidosis. Affected family members developed nephropathy almost three decades later and five heterozygous carriers developed hypertension in their forties and fifties. Thus, the clinical phenotype of our homozygous patient was more severe (earlier onset of nephropathy, cardiac and hepatic involvement) than those of heterozygotes in the same family, consistent with gene dosage effects on the phenotype of AFib amyloidosis. The follow-up of hypertensive heterozygous carriers will be helpful in the study of the pathogenesis of hypertension in AFib amyloidosis.

In systemic amyloidosis, solid organ transplantation has been used to replace failing organ function [26–29]. Isolated RTx alone has been performed for ESRF in several patients with AFib and probably remains appropriate when there is good evidence that amyloid deposition does not threaten the function of other vital organs. Gillmore et al. reported that RTx in AFib is associated with recurrence of amyloid in the graft with resultant loss of transplanted kidneys after a median of 6.7 years [3]. CLKT in a patient with amyloidotic renal failure caused by the* FGA* p.Glu545Val mutation was first performed in 1995 [28]. Despite liver transplantation being the only currently available curative treatment for AFib, it seems reasonable to propose it for younger and fitter patients, weighing the high risk of early perioperative death following CLKT against the elimination of the risk of recurrent amyloid disease in the allograft [3, 29]. Transplantation in our patient aimed to replace her failing organ function, because in 1996 her amyloid type was unknown; it was 5 years later that we made a retrospective diagnosis of hereditary fibrinogen amyloidosis. Despite isolated RTx, our patient's outcome was not unfavourable compared to the results with CLKT [3, 4]. Her apparent cardiac and hepatic AFib involvement progressed despite clinical absence of disease recurrence in the allograft. Mild proteinuria appeared 13 years after transplantation, but allograft function remained good until 4 months before the patient's death. At that time, cardiovascular symptoms were her principal problem, due to cardiac amyloidosis suggested by echocardiography. In patients with cardiac AFib involvement, combined heart-liver, heart-kidney, or heart-liver-kidney transplantation could be discussed [30, 31], but, given the high risk in our 67-year-old patient, she received recommended drug treatment only. She died 16 years after renal transplantation due to severe heart failure in the context of sepsis.

## 4. Conclusions

In conclusion, correct identification of amyloidogenic protein should always be pursued, even retrospectively, because it enables the choice of the most appropriate therapy, avoids unnecessary and potentially harmful treatments, and ensures family screening. This first report of a homozygous AFibE526V (p.Glu545Val) amyloidosis expands our knowledge about the phenotype and the outcome of isolated renal transplantation and may be relevant for understanding the molecular mechanisms of dominance in hereditary amyloidosis.

## Figures and Tables

**Table 1:** Homozygous amyloidogenic variants reported in the literature.

Gene	Protein variant	Sequence variant (mRNA)	Patients (*n*)	Geographic origin/ethnicity	Reported phenotype	Clinical course	References
*GSN *	Asp187Asn (p.Asp214Asn)	c.640G>A	2	Finland	CN, CLD, SC, CRF	Severe nephropathy	[12, 13]

*TTR *	Val30Met (p.Val50Met)	c.148G>A	19	Japan Spain Sweden Turkey	PN, AN, VO, GI, H, CN	Wide variability, from asymptomatic carriers to slightly more severe phenotypes with higher incidence rate and earlier onset than heterozygotes within the same family	[14–19]

	Leu58His (p.Leu78His)	c.233T>G	1	American/German	PN, CMP	More rapid course of disease	[20]

	Phe64Leu (p.Phe84Leu)	c.250T>C	1	Italy	PN, AN, CMP	More severe phenotype	[21]

	Val122Ile (p.Val142Ile)	c.424G>A	24	African/American	CMP	Earlier age at onset and uncertain penetrance, particularly with respect to gender	[22, 23]

AN: autonomic neuropathy; CLD: corneal lattice dystrophy; CMP: cardiomyopathy; CN: cranial neuropathy; CRF: chronic renal failure; GI: gastrointestinal symptoms; H: heart conduction disturbance; PN: peripheral polyneuropathy; SC: skin changes; VO: vitreous opacities.

**Table 2:** Laboratory data.

Parameter/date	02.14.2006	04.07.2009	08.17.2010	08.14.2012	11.26.2012	12.05.2012
Urea (mg/dL)	69	41	89	136	135	188
Creatinine (mg/dL)	0.80	0.75	1.03	1.22	1.44	4.62
Creatinine clearance (mL/min/1.73 m^2^)	67.9	84.7	68.3	45.9	37.6	9.2
Albumin (g/L)	43.0		45.7			
HbA1c (%)			5.9			
Uric acid (mg/dL)		6.7	8.9	7.0		
Total bilirubin (mg/dL)	0.79	0.60	0.60	0.72	1.13	0.96
AST (U/L)	21	23	23	19	14	22
ALT(U/L)	19	14	20	15	2	12
ALP (U/L)	102	106	134	139	160	170
GGT (U/L)	90	86	88	81	106	65
Sodium (mmol/L)	144		134	136	132	122
Potassium (mmol/L)	4.73		4.17	4.50	4.87	5.73
Chloride (mmol/L)	106		96	101	96	98
Calcium (mmol/L)	2.52		2.56			2.02
Phosphorus (mmol/L)	1.03		1.18			2.09
iPTH (pg/mL)	75	80	103			
Total cholesterol (mg/dL)	203	207	196	155		
Triglycerides (mg/dL)	127	78	105	111		
NT-proBNP (pg/mL)					14 056	
CRP (mg/dL)						85.62
Cyclosporine (ng/mL)		80.5	94.7			130.2
Proteinuria (g/24 h)	0.15	0.90	0.51			

HbA1c: glycated hemoglobin; AST: aspartate aminotransferase; ALT: alanine aminotransferase; ALP: alkaline phosphatase; GGT: gamma-glutamyl transferase; iPTH: intact parathyroid hormone; NT-proBNP: N-terminal pro-B-type natriuretic peptide; CRP: C-reactive protein.
